# Continuity of Care, Follow-Up Care, and Outcomes among Breast Cancer Survivors

**DOI:** 10.3390/ijerph16173050

**Published:** 2019-08-22

**Authors:** Yun-Yi Chen, Cheng-I Hsieh, Kuo-Piao Chung

**Affiliations:** 1 Institute of Health Policy and Management, College of Public Health, National Taiwan University, Taipei 10055, Taiwan; 2 Division of Hematology and Oncology, Department of Internal Medicine, Taipei Medical University Hospital, Taipei 11031, Taiwan; 3 Division of Hematology and Oncology, Department of Internal Medicine, School of Medicine, College of Medicine, Taipei Medical University, Taipei 11031, Taiwan

**Keywords:** breast cancer, survivors, continuity of care, care continuity, follow-up care, surveillance, health outcome, hospitalization, emergency department use

## Abstract

This retrospective cohort study examined the effects of care continuity on the utilization of follow-up services and outcome of breast cancer patients (stages I–III) in the post-treatment phase of care. Propensity score matching and generalized estimation equations were used in the analysis of data obtained from national longitudinal databases. The continuity of care index (COCI) was calculated separately for primary care physicians (PCP) and oncologists. Our results revealed that breast cancer survivors with a higher oncology COCI were more likely than those with a lower oncology COCI to use mammography or breast ultrasound during the follow-up period (OR = 1.26, 95% CI: 1.19–1.32; OR = 1.12, 95% CI: 1.06–1.18; respectively). In terms of health outcomes, a higher oncology COCI was associated with a lower likelihood of hospitalization (OR = 0.78, 95% CI: 0.71–0.85) and emergency department use (OR = 0.88, 95% CI: 0.82–0.95). A higher PCP COCI was also associated with a lower likelihood of hospitalization (OR = 0.77, 95% CI: 0.70–0.85) and emergency department use (OR = 0.75, 95% CI: 0.68–0.82). Overall, this study determined that ambulatory care continuity is positively associated with the likelihood of using recommended follow-up care services and negatively associated with adverse health events among breast cancer survivors.

## 1. Introduction

Early diagnosis and improvements in cancer treatment have greatly enhanced the likelihood of cancer survival, and the number of cancer survivors is expected to reach 20 million by 2026 [1]. Since the National Institute of Medicine (IOM) reported on the importance of survivorship care [2], this issue has gradually attracted attention. Survivorship care has even been described as a paradigm shift in the cancer care continuum [3]. The follow-up period after cancer treatment is a distinct phase of care, involving psychosocial, community and supportive care, health promotion, regular monitoring, and long-term follow-up as well as interventions for late-effects [4,5,6].

Cancer care is a worldwide problem of considerable complexity and fragmentation [7,8]. The fact that treatment can have a major impact on the long-term health and quality of life of survivors greatly complicates disease management, and the disease burden of survivors is often underestimated. Many patients with limited resources must deal with intermittent healthcare and compromised adherence to treatment regimes. In some cases, regular surveillance care in accordance with established guidelines is underused [9,10]. In other cases, advanced imaging diagnostics (widely regarded as low-value care), are overused [9,11,12,13]. The American Society of Clinical Oncology (ASCO) has listed the overuse of advanced imaging methods for breast cancer as one of the top five issues that must be addressed in efforts to improve the quality of cancer care and reduce the associated costs [14]. 

Continuity of care is concerned with the quality of care over time as well as fairness and efficiency [15,16,17,18,19,20,21,22,23]. Some empirical studies have surveyed care continuity from the perspective of cancer patients [23,24,25]. Previous research has indicated that higher care continuity is associated with a stronger sense of satisfaction, higher quality of life, and better mental health [19,22]. Poor care continuity is associated with excessive consumption of medical resources, primarily through the consumption of unnecessary services [26]. 

One population-based study linked a lower oncology care continuity to a higher likelihood that breast cancer survivors would exceed the recommended number of visits to their oncologist [27]; however, there has been little research on the relationship between care continuity and health outcomes among cancer survivors empirically by using claims-based measurements. In addition, Taiwan has a unique medical environment which might undermine the care continuity [28]. The implementation of universal health insurance has greatly improved access to medical treatment in Taiwan; however, the family physician arrangement has not been formally implemented. This means that under the current scheme, patients may visit specialists without a referral [29].

Breast cancer is the most common form of cancer among women; however, the survival rate is relatively high [30]. Breast cancer survivors face multiple medical and psychosocial needs as they progress from treatment to survival [31]. Nonetheless, it has been shown that many breast cancer survivors do not receive adequate care [32,33,34,35]. Therefore, we employed a nationally representative longitudinal database from the universal health insurance program in Taiwan to evaluate the effects of post-treatment care continuity on follow-up care utilization and health outcomes among breast cancer survivors in this study.

## 2. Methods 

### 2.1. Study Design, Data Resource, Participants

This retrospective cohort study was based on the long-form databases of the Taiwan Cancer Registry, comprising a nationally representative cohort of patients diagnosed with cancer [36,37]. Patients newly diagnosed with breast cancer between January 1, 2002 and December 31, 2007 were included. Follow-up information extending until December 31, 2012 was obtained via data linkage using profiles from the National Healthcare Insurance Database (NHIRD) and the National Register of Deaths. Data resources were collected, organized, and managed by the Health and Welfare Data Science Center (HWDC) of the Ministry of Health and Welfare (MOHW). The index date for each patient in this study was set at 366 days post-diagnosis. The follow-up period was defined as the period from one year after the index date to four years after the index date (or sooner if censored), as this covers the period in which survivors are followed-up most intensively [27]. The recurrence or death during the follow-up period was considered as censored. This study was approved by the Institutional Review Board at the National Taiwan University Hospital (IRB approval number 201405054w), which waived the requirement for informed consent.

**Figure 1:**
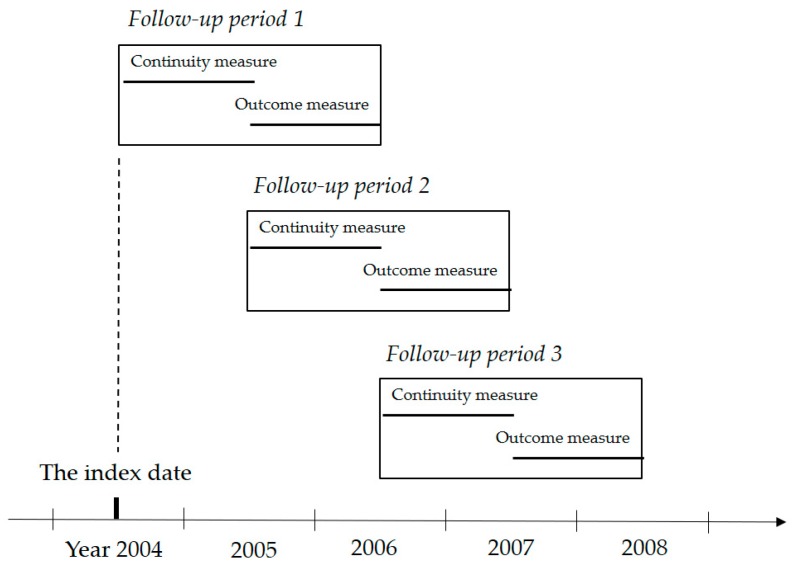
Study design.

In accordance with previous studies [38,39,40,41], patients were selected for inclusion based on the following criteria: (1) newly diagnosed with breast cancer, (2) confirmed diagnosis of cancer between 2002 and 2007, (3) diagnosis of Stage I–III breast cancer, (4) >20 years old at the time of diagnosis, and (5) survived at least two years after diagnosis. Exclusion criteria included (1) missing information including age, gender, and date of cancer diagnosis date, and (2) receiving chemotherapy or radiation therapy during the follow-up period. Additionally, our focus in this study was on care continuity in the outpatient setting, and particularly in the second year after diagnosis during which survivors switch from treatment to follow-up care. Thus, cases without any outpatient records beyond the second year after diagnosis were also excluded. In order to avoid the problem of reverse causality, the care continuity was measured for one-year period and the measurement of follow-up care and health outcome was measured for a subsequent period (Figure 1).

### 2.2. Variables

#### 2.2.1. Independent Variables

The independent variable was continuity of care between patients and their physician (solely for outpatient services) during the follow-up period. We adopted the continuity of care index (COCI) to calculate care continuity due to the larger number of physician visits typical of the Taiwanese healthcare system. The COCI derived from the number of different physicians visited and the number of visits made to each physician [42]. Note that this index is less sensitive to the number of visits to physicians [43].

The equation used to derive the index is as follows:

(1) $$COCI=\frac{\sum_{j=1}^{M}n_{j}{}^{2}-N}{N(N-1)}$$,

where *N* represents the total number of primary care physician visits for a given patient, *n_j_* is the number of visits to the same physician j, and M is the total number of physicians for a given patient. COCI was calculated separately for primary care physicians (PCP) and oncology specialists. PCPs were physicians dealing with general medicine, internal medicine, family medicine, and obstetrics/gynecology [44,45,46]. Oncology specialists included subspecialists in medical oncology, hematology oncology, surgeons, or radiation oncologists. 

COCI was calculated only for patients who made at least three visits, due to the fact that continuity of care would be meaningless if based a small number of visits [47,48,49]. COCI is bounded between 0 and 1, where a higher value indicates greater continuity of care. The value assigned for continuity of care has no inherent clinical meaning; therefore, we divided the sample into two groups (high and low continuity) based on the median COCI and distribution of scores across the entire study population. COCI was treated as time-varying variable for each year.

#### 2.2.2. Dependent Variables

Measurements of follow-up care included the use of annual surveillance mammograms, breast ultrasounds, and advanced imaging tests for metastatic disease, which included chest X-rays, bone scans, liver ultrasound, computed tomography (CT) scans, positron emission tomography (PET) scans, and breast magnetic resonance imaging (MRI). The health outcomes included instances of hospital admission or emergency department visit in a given year. All outcome measures were coded as dichotomous variables.

#### 2.2.3. Covariates

Patient characteristics included the year of diagnosis, age at the time of diagnosis, tumor stage, hormone receptor status, type of surgery, health status, occupation, and the level of insurance premiums. We used the modified Charlson Comorbidity Index (CCI) [50], the number of visits to physicians, and the likelihood of hospitalization for any reason in the year prior to breast cancer diagnosis as proxy variables for a patient health status. Modified CCI values were used to recalculate the score excluding cancer-related diagnoses. Regional characteristics included the level of urbanization and the number of physicians per square kilometer in the area of residence. The characteristics of the medical provider most frequently visited by the patient each year included the age of the physician and the average annual breast cancer volume of the physician. The characteristics of the medical institution most frequently visited by the patient each year included the accreditation level, ownership, and average annual breast cancer volume of the facility.

### 2.3. Statistical Analysis

In cases of bivariate analysis, the chi-square test was used for the analysis of categorical variables. The continuous variable was first checked using the Kolmogorov–Smirnov test for normality. If the data presented normal distribution, then the Student’s t-test was used; otherwise, the Mann–Whitney U Test was used. Additionally, we used propensity score matching (PSM) to minimize confounding effects [51]. In estimating propensity scores for oncology care continuity and PCP care continuity, the variables used in the logistic regression models included patient characteristics (year of diagnosis, age, tumor stage, hormone receptor status, type of surgical procedure, health status, and socioeconomic variables), regional characteristics (urbanization and number of physicians per square kilometer in area of patient residence), physician characteristics (age, average annual breast cancer volume), and the hospital characteristics (accreditation level, ownership, and average annual breast cancer volume).

Data were randomized using nearest neighbor matching, matching without replacement, and the tolerable caliper width was set at 0.001. After matching, the sample was analyzed in terms of standardized difference to determine whether the distribution of data was balanced. Generalized estimating equations (GEEs) were used for data analysis in order to control for subject characteristics that were not observed during the study, such as healthcare-seeking behavior. Note that GEE methods are commonly used for the analysis of correlated data to obtain unbiased estimates of coefficients despite possible misspecification of the correlation structure [52]. To avoid over-adjustment, we opted not to include in our analysis any variables that had been used in propensity score matching and had been balanced after matching. 

## 3. Results

### 3.1. Patient Characteristics

This study included patients newly diagnosed with breast cancer (Stage I, II, or III) between 2002 and 2007. A total of 18,031 patients were included in the analysis. Table 1 presents the distribution of baseline characteristics of the sample grouped according to the continuity of oncology care. Among breast cancer survivors with higher continuity of oncology care, the average age at the time of diagnosis was 51.7 years with the age distribution peaking at 45–54 years (37%), followed by 35–44 years old (22%), and 55–64 years (21%). In terms of clinical characteristics, most of the tumors were in Stage II (48%), followed by Stage I (34%). The estrogen receptor (ER)-positive rate was 52%, and progesterone receptor (PR)-positive rate was 47%. Approximately 25% of the patients underwent breast conserving surgery (BCS). In terms of basic health status, the CCI score of most of the patients was 0 (80%) or 1 (13%), and 16% were hospitalized one year prior to diagnosis. The occupation of most of the patients fell within the category of labor (31%). It was observed that 2% of the patients were physicians or family members of physicians. Most of the patients resided in urban areas (69%). The facilities most frequently visited by patients in the second year after diagnosis were medical centers (55%), most of which were non-public hospitals (80%). The physicians most frequently visited by patients were 40–59 years old (74%). Following PSM, the distributions of the variables were balanced.

Table 2 presents the distribution of baseline characteristics of the sample grouped according to continuity of care by primary physicians (PCP). At the time of diagnosis, the average age of breast cancer survivors experiencing high continuity of primary care was 54.9 years with the age distribution peaking at 45–54 years (34%) followed by 55–64 years (26%). In terms of clinical characteristics, most of the tumors were in Stage II (47%) followed by Stage I (34%). The proportion of ER-positive results was 47%, and the proportion of PR-positive results was 42%. BCS accounted for 28% of the surgical procedures. In terms of basic health status, most of the patients had CCI scores of 0 (72%) or 1 (20%), and 16% were hospitalized during the year prior to diagnosis. Most of the patients were unemployed (28%) or categorized as labor (27%). It was observed that 1.6% of the patients were physicians or family members of physicians. Most of the patients resided in urban areas (65%). The hospitals most frequently visited in the second year after diagnosis were medical centers (66%), most of which were non-public hospitals (81%). The physicians most frequently visited by patients were between 40–59 years (74%). Following PSM, the distributions of the variables were balanced.

### 3.2. Distribution of Care Continuity, Follow-Up Care, and Health Outcome

Table 3 presents the values of ambulatory care continuity, the percentage of follow-up service utilization, and negative health outcomes in the various follow-up periods. The average continuity of oncology care value was 0.70–0.72, and the average continuity of PCP care value was 0.52–0.54. In terms of follow-up services, 56–57% of the breast cancer survivors underwent mammography and 73–75% underwent breast ultrasound. Advanced imaging tests were administered to 82–85% of the patients. In terms of health outcomes, 11–13% of the patients were hospitalized and 12–15% visited the emergency department in each of the years (Table 3).

### 3.3. Effect of Oncology Care Continuity and PCP Care Continuity

**Figure A1:**
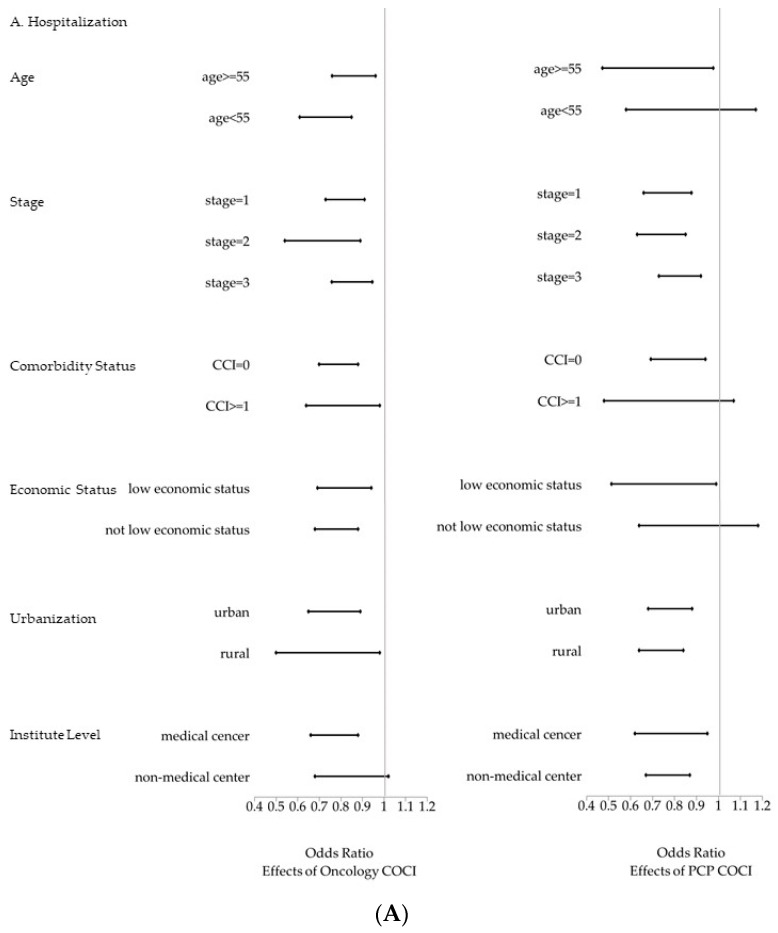

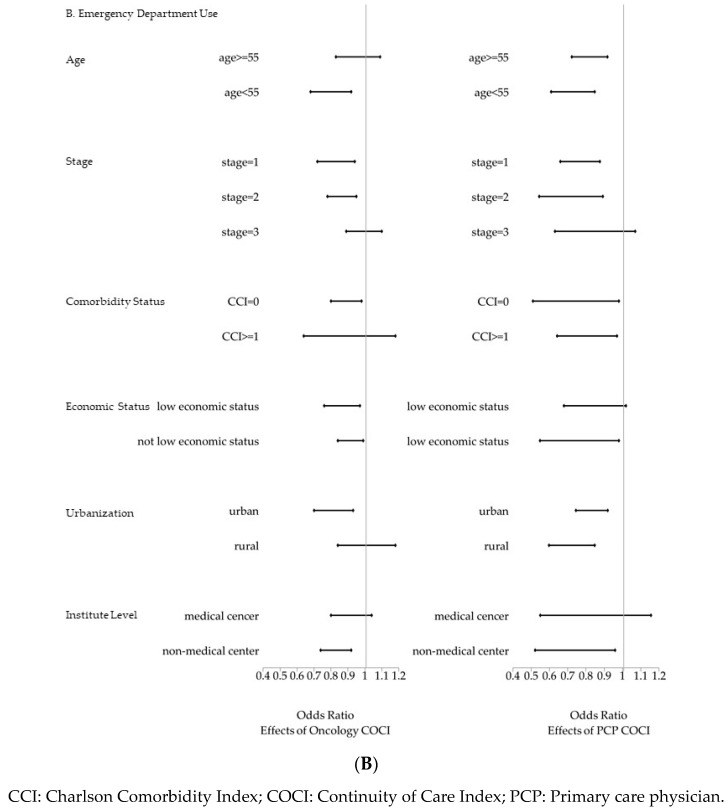
Subgroup analysis of odds ratio between the high COCI and low COCI groups for health events of (**A**) all-cause hospitalization and (**B**) all-cause emergency department use.

Table 4 lists sample analysis after PSM. The distribution of each variable was balanced; therefore, we did not include the covariates used for adjustment; i.e., the table presents crude odds ratios). In terms of follow-up care utilization, breast cancer survivors with a higher oncology COCI was more likely to use mammography (OR = 1.26, 95% CI: 1.19–1.32), breast ultrasound (OR = 1.12, 95% CI: 1.06–1.18), or advanced imaging tests (OR = 1.47, 95% CI: 1.36–1.60). Primary care COCI was not associated with the use of these services. In terms of health outcomes, a higher oncology COCI was associated with a lower likelihood of hospitalization (OR = 0.78, 95% CI: 0.71–0.85) and lower likelihood of emergency department use (OR = 0.88, 95% CI: 0.82–0.95). Similarly, a higher primary care COCI was associated with lower likelihood of hospitalization (OR = 0.77, 95% CI: 0.70–0.85) and lower likelihood of emergency department use (OR = 0.75, 95% CI: 0.68–0.82). In addition, we used subgroup analysis to explore the potential effects of other variables on the association between continuity of care and the likelihood of adverse health events. The results are presented in Appendix A, Figure A1.

## 4. Discussion

This study examined the correlations between ambulatory continuity of care (by oncologists and primary care physicians) and the utilization of follow-up care by breast cancer survivors and their health outcomes.

Existing guidelines suggest that breast cancer survivors regularly undergo mammography in the first five years of the follow-up period; however, only 56%–57% of breast cancer survivors in this study actually undergo annual mammography during that period. The use of mammography by breast cancer survivors was estimated at 41% in South Korea [53], 54% in the UK [34], and 47%–82% in the US [9,44,46,54,55]. Considering the fear of pain caused by mammography and the higher density of breast tissue in Asian women, it is not uncommon for clinicians to opt for breast ultrasound as an alternative for follow-up monitoring. We discovered that 80% of the breast cancer survivors underwent mammography or breast ultrasonography in each year of the follow-up period. 

On the other hand, we observed that 80% of breast cancer survivors used advanced imaging tests that were not recommended in current guidelines. Based on a review of medical records, Hahn et al. reported that 55% of breast cancer survivors in the US underwent at least one imaging examination that was not recommended for the monitoring of cancer status [22]. Note that we were unable to confirm the purpose of using the various examinations with administrative datasets; therefore, the relevant proportion may be overestimated. This type of imaging test exposes the patient to unnecessary high doses of radiation and many imaging tests are prone to false positive results, which can lead to anxiety and in some cases unnecessary invasive treatment [14]. In one study, it was surmised that the use of unnecessary imaging tests can be attributed to requests from patients for more aggressive examinations [56].

In the current study, the average ambulatory COCI values were as follows: oncology specialists (0.71) and primary care physicians (0.54). In relevant empirical studies on chronic diseases in the past, the mean COCI of outpatient care provided by physicians was 0.66 for asthma patients [57], 0.55–0.79 for patients with chronic obstructive pulmonary disease [57,58,59], 0.33–0.55 for patients with chronic heart failure [60], 0.50–0.71 for patients with diabetes [58], and 0.74 for patients with hypertension [57]. Continuity of care is related to disease characteristics; therefore, patients requiring long-term treatment and follow-up tend to have better ambulatory continuity of care [59]. The higher COCI values observed in this study may correlate with the risk of recurrence and the requirements of health management (e.g., dealing with late-effect symptoms). Anxiety about recurrence often prompts breast cancer survivors to seek follow-up support from oncologists [61].

Our results revealed that patients who continued to receive care from their oncologist were more likely to use mammography and/or breast ultrasonography, whereas patients who received follow-up care from their primary care physician were not likely to do so. Additionally, our results indicate that continuity of care, no matter for oncologists or for PCP, is negatively associated with hospitalization and emergency department visits. This result is consistent with the influence direction reported in previous studies [20,62,63,64,65,66]. Continuity of care can promote a willingness on the part of patients to communicate disease-related information. It also makes it easier for patients and their families to deal with the disease. It appears that continuity of care can alter the health behavior of patients [67]. A good physician–patient relationship may increase the likelihood that the patient will seek medical help before the condition becomes urgent, thereby reducing the possibility of hospital admission or emergency department use. Long-term physician–patient relationships also make it easier for the physician to understand changes in the health status and needs of the patient. Patients who feel trust and satisfaction with their physician are more likely to comply with disease management instructions, such as compliance in taking medication [68]. At the provider level, regular care is highly conducive to information continuity. Lack of care continuity has been associated with a higher number of medical errors, such as duplicate medication and inappropriate medication [49,69,70].

This study has several limitations. First, the secondary database used in this study did not include clinical test values or data pertaining to comprehensive health status (e.g., awareness and physiological functions), socio-economic status (e.g., education level and actual income), health literacy, social network, psychological support resources, diet or exercise, or the life style choices of breast cancer survivors. We were also unable to confirm the care decision-making process, which made it impossible to determine whether the use of care services was related to the attitudes of health care providers or patient preferences. Furthermore, our use of the National Health Insurance Research Database made it impossible to obtain relevant information on self-paid health examinations from other archives. We were unable to confirm the reasons for using the various imaging tests; i.e., it is difficult to distinguish between surveillance and diagnostic procedures. This increases the likelihood that we overestimated the use of low-value (non-recommended) imaging diagnostics. Third, the indicators of continuity of care adopted in this study reflect only the degree to which patients sought medical care from regular medical providers. Thus, it was not possible for us to examine the variables related to physician–patient relationships, communication, and trust. Our analysis was also limited to physicians, such that the characteristics of the entire medical team could not be taken into consideration. Finally, it is possible that the influence of continuity of care may vary over time. This issue could be addressed in future studies. 

## 5. Conclusions

This study provides evidence that breast cancer survivors receiving ambulatory care continuity are more likely to use recommended surveillance care services and less likely to experience negative health events. Maintaining regular source of care should be adequately addressed in the post-treatment phase of cancer care.

## Figures and Tables

**Table 1:** Baseline characteristics of patients grouped according to the oncology continuity of care.

Characteristics	Pre-PSM Sample ^a^					Post-PSM Sample					
	Low COCI (*n* = 7133, 50%)		High COCI (*n* = 7129, 50%)		*p* Value	Low COCI (*n* = 5545, 50%)		High COCI (*n* = 5545, 50%)		*p* Value	Standardized Difference
	*n*	%	*n*	%		*n*	%	*n*	%		
Year of diagnosis					0.0064					0.9913	0.005
2002	274	3.84	219	3.07		202	3.64	194	3.50		
2003	379	5.31	334	4.69		281	5.07	274	4.94		
2004	1304	18.28	1363	19.12		1035	18.67	1034	18.65		
2005	1447	20.29	1568	21.99		1185	21.37	1189	21.44		
2006	1706	23.92	1680	23.57		1316	23.73	1340	24.17		
2007	2023	28.36	1965	27.56		1526	27.52	1514	27.30		
Age of diagnosis					<0.0001					0.9199	–0.005
<35	326	4.57	310	4.35		244	4.40	254	4.58		
35–44	1765	24.74	1597	22.40		1293	23.32	1306	23.55		
45–54	2714	38.05	2662	37.34		2115	38.14	2081	37.53		
55–64	1452	20.36	1508	21.15		1131	20.4	1139	20.54		
65–74	654	9.17	790	11.08		563	10.15	581	10.48		
75+	222	3.11	262	3.68		199	3.59	184	3.32		
Stage					0.0010					0.9819	0.001
I	2469	34.61	2446	34.31		1925	34.72	1918	34.59		
II	3222	45.17	3398	47.66		2552	46.02	2562	46.20		
III	1442	20.22	1285	18.02		1068	19.26	1065	19.21		
ER					0.0219					0.9362	–0.006
Negative	1417	19.87	1307	18.33		1058	19.08	1073	19.35		
Positive	3541	49.64	3685	51.69		2835	51.13	2827	50.98		
Unknown	2175	30.49	2137	29.98		1652	29.79	1645	29.67		
PR					0.0004					0.876	0.002
Negative	1802	25.26	1625	22.79		1343	24.22	1326	23.91		
Positive	3156	44.25	3362	47.16		2549	45.97	2575	46.44		
Unknown	2175	30.49	2142	30.05		1653	29.81	1644	29.65		
Type of surgery					<0.0001					0.548	–0.011
BCS	2590	36.31	1784	25.02		1680	30.3	1651	29.77		
Else	4543	63.69	5345	74.98		3865	69.7	3894	70.23		
CCI score					0.1164					0.7611	–0.009
0	5661	79.36	5730	80.38		4418	79.68	4429	79.87		
1	968	13.57	955	13.40		749	13.51	757	13.65		
2+	504	7.07	444	6.23		378	6.82	359	6.47		
Prior hospitalization					0.1088					0.6401	0.009
Yes	1083	15.18	1152	16.16		872	15.73	890	16.05		
No	6050	84.82	5977	83.84		4673	84.27	4655	83.95		
Number of outpatient visits					<0.0001					0.9904	0.000
Low	1808	25.35	2077	29.13		1521	27.43	1518	27.38		
Median	2491	34.92	2620	36.75		1964	35.42	1971	35.55		
High	2834	39.73	2432	34.11		2060	37.15	2056	37.08		
Level of insurance premiums					<0.0001					0.9574	–0.009
Low	1523	21.35	1552	21.77		1205	21.73	1212	21.86		
Mid-Low	1910	26.78	2120	29.74		1544	27.84	1560	28.13		
Mid-High	1489	20.87	1493	20.94		1153	20.79	1156	20.85		
High	2211	31.00	1964	27.55		1643	29.63	1617	29.16		
Occupation status					0.0002					0.8437	0.004
Labor	2362	33.11	2187	30.68		1727	31.15	1768	31.88		
Public servant	1868	26.19	1905	26.72		1510	27.23	1474	26.58		
Farmer or fishermen	861	12.07	1018	14.28		714	12.88	723	13.04		
Low-income households ^b^	64	0.90	48	0.67		57	1.03	43	0.78		
Unemployed	1978	27.73	1971	27.65		1537	27.72	1537	27.72		
Whether physicians/family members of physicians					0.8187					0.7476	–0.006
Yes	151	2.12	147	2.06		126	2.27	121	2.18		
No	6982	97.88	6982	97.94		5419	97.73	5424	97.82		
Urbanization					<0.0001					0.9834	0.000
Low	1972	27.65	2207	30.96		1638	29.54	1639	29.56		
High	5161	72.35	4922	69.04		3907	70.46	3906	70.44		
Number of physicians per square kilometer					<0.0001					0.8113	–0.008
Low	3357	47.07	3612	50.67		2717	49.00	2754	49.67		
High	3776	52.94	3517	49.33		2828	51.00	2791	50.33		
Age of physician					0.0002					0.9917	0.001
<40	1553	21.77	1439	20.19		1162	20.96	1157	20.87		
40–59	5132	71.95	5251	73.66		4056	73.15	4062	73.26		
60+	448	6.28	439	6.16		327	5.90	326	5.88		
Average annual breast cancer volume of the physician					<0.0001					0.8578	–0.008
Low	3972	55.68	3145	44.12		2789	50.30	2801	50.51		
Median	1383	19.39	2198	30.83		1299	23.43	1312	23.66		
High	1778	24.93	1786	25.05		1457	26.28	1432	25.83		
Accreditation level					0.0595					0.8045	0.005
Medical center	3799	53.26	3909	54.83		2962	53.42	2975	53.65		
Non-medical center	3334	46.74	3220	45.17		2583	46.58	2570	46.35		
Ownership					<0.0001					0.5364	–0.012
Public hospital	2192	30.73	1423	19.96		1372	24.74	1344	24.24		
Non-public hospital	4941	69.27	5706	80.04		4173	75.26	4201	75.76		
Average annual breast cancer volume of the facility					<0.0001					0.7792	–0.012
Low	3179	44.57	3506	49.18		2618	47.21	2638	47.57		
Median	2065	28.95	2231	31.29		1669	30.10	1680	30.30		
High	1889	26.48	1392	19.53		1258	22.69	1227	22.13		

PSM: propensity score matching; COCI: continuity of care index; ER: estrogen receptor; PR:progesterone receptor; CCI: Charlson comorbidity index; BCS: breast cancer surgery. ^a^ The analytic sample included only the patients who made at least 3 visits for oncologists in the second year after diagnosis. ^b^ Insured income is lower than the level required for charging premium.

**Table 2:** Baseline characteristics of patients grouped according to the PCP continuity of care.

Characteristics	Pre-PSM Sample ^a^					Post-PSM Sample					
	Low COCI (*n* = 3058, 49%)		High COCI (*n* = 3143, 51%)		*p* Value	Low COCI (*n* = 2565, 50%)		High COCI (*n* = 2565, 50%)		*p* Value	Standardized Difference
	*n*	%	*n*	%		*n*	%	*n*	%		
Year of diagnosis					0.0025					0.9954	0.006
2002	122	3.99	123	3.91		100	3.9	106	4.13		
2003	212	6.93	196	6.24		164	6.39	164	6.39		
2004	652	21.32	625	19.89		526	20.51	530	20.66		
2005	728	23.81	662	21.06		583	22.73	579	22.57		
2006	624	20.41	670	21.32		546	21.29	533	20.78		
2007	720	23.54	867	27.59		646	25.19	653	25.46		
Age of diagnosis					0.0683					0.9942	0.001
<35	85	2.78	70	2.23		66	2.57	62	2.42		
35–44	507	16.58	507	16.13		412	16.06	415	16.18		
45–54	1016	33.22	1065	33.88		851	33.18	862	33.61		
55–64	727	23.77	830	26.41		650	25.34	634	24.72		
65–74	514	16.81	473	15.05		416	16.22	421	16.41		
75+	209	6.83	198	6.30		170	6.63	171	6.67		
Stage					0.0961					0.7737	0.019
I	1114	36.43	1075	34.20		913	35.59	896	34.93		
II	1421	46.47	1478	47.03		1202	46.86	1201	46.82		
III	523	17.10	590	18.77		450	17.54	468	18.25		
ER					0.0028					0.871	0.015
Negative	761	24.89	705	22.43		607	23.66	594	23.16		
Positive	1482	48.46	1485	47.25		1240	48.34	1239	48.3		
Unknown	815	26.65	953	30.32		718	27.99	732	28.54		
PR					0.0034					0.7007	0.021
Negative	906	29.63	855	27.21		742	28.93	715	27.88		
Positive	1337	43.72	1333	42.41		1104	43.04	1117	43.55		
Unknown	815	26.65	955	30.38		719	28.03	733	28.58		
Type of surgery					0.7421					0.9007	0.003
BCS	855	27.96	867	27.59		713	27.8	709	27.64		
Else	2203	72.04	2276	72.41		1852	72.2	1856	72.36		
CCI score					0.0019					0.973	0.007
0	2107	68.90	2261	71.94		1794	69.94	1801	70.21		
1	629	20.57	629	20.01		523	20.39	520	20.27		
2+	322	10.53	253	8.05		248	9.67	244	9.51		
Prior hospitalization					0.0755					0.5019	0.019
Yes	537	17.56	499	15.88		441	17.19	423	16.49		
No	2521	82.44	2644	84.12		2124	82.81	2142	83.51		
Number of outpatient visits					<0.0001					0.7804	0.017
Low	843	27.57	1056	33.60		763	29.75	774	30.18		
Median	1081	35.35	1100	35.00		901	35.13	914	35.63		
High	1134	37.08	987	31.40		901	35.13	877	34.19		
Level of insurance premiums					0.0009					0.909	0.008
Low	768	25.11	777	24.72		665	25.93	655	25.54		
Mid-Low	749	24.49	691	21.99		593	23.12	604	23.55		
Mid-High	823	26.91	803	25.55		678	26.43	662	25.81		
High	718	23.48	872	27.74		629	24.52	644	25.11		
Occupation status					0.0018					0.9864	0.010
Labor	719	23.51	850	27.04		619	24.13	634	24.72		
Public servant	775	25.34	799	25.42		654	25.50	643	25.07		
Farmer or fishermen	664	21.71	575	18.29		510	19.88	506	19.73		
Low-income households ^b^	35	1.14	41	1.30		35	1.36	33	1.29		
Unemployed	829	27.94	914	28.26		747	29.12	749	29.20		
Whether physicians/family members of physicians					0.1581					0.8071	0.007
Yes	35	1.14	49	1.56		33	1.29	35	1.36		
No	3023	98.86	3094	98.44		2532	98.71	2530	98.64		
Urbanization					0.0610					0.7276	0.010
Low	1151	37.64	1111	35.35		938	36.57	926	36.10		
High	1907	62.36	2032	64.65		1627	63.43	1639	63.90		
Number of physicians per square kilometer					0.2812					0.8736	0.014
Low	1554	50.82	1529	48.65		1266	49.35	1270	49.52		
High	1504	49.18	1614	51.35		1299	50.65	1295	50.48		
Age of physician					<0.0001					0.899	0.012
<40	645	21.09	550	17.50		509	19.84	502	19.57		
40–59	2202	72.01	2313	73.59		1865	72.71	1864	72.67		
60+	211	6.90	280	8.91		191	7.45	199	7.76		
Average annual breast cancer volume of the physician					<0.0001					0.8736	0.014
Low	1370	44.80	1731	55.07		1271	49.55	1289	50.25		
Median	746	24.40	799	25.42		672	26.2	666	25.96		
High	942	30.80	613	19.50		622	24.25	610	23.78		
Accreditation level					<0.0001					0.9082	0.003
Medical center	1255	41.04	1069	34.01		964	37.58	960	37.43		
Non-medical center	1803	58.96	2074	65.99		1601	62.42	1605	62.57		
Ownership					<0.0001					0.6116	0.014
Public hospital	730	23.87	608	19.34		550	21.44	565	22.03		
Non-public hospital	2328	76.13	2535	80.66		2015	78.56	2000	77.97		
Average annual breast cancer volume of the facility					<0.0001					0.9726	0.003
Low	1399	45.75	1692	53.83		1269	49.47	1269	49.47		
Median	851	27.83	766	24.37		674	26.28	680	26.51		
High	808	26.42	685	21.79		622	24.25	616	24.02		

PCP: primary care physician; PSM: propensity score matching; COCI: continuity of care index; ER: estrogen receptor; PR: progesterone receptor; CCI: Charlson Comorbidity Index; BCS: breast cancer surgery. ^a^ The analytic sample included only the patients who made at least three visits for primary care physicians in the second year after diagnosis. ^b^ Insured income is lower than the level required for charging premium.

**Table 3:** Summary statistics for main interest variables according to follow-up period.

Variable	Follow-Up Period 1 (*n* = 18031)	Follow-Up Period 2 (*n* = 16904)	Follow-Up Period 3 (*n* = 15990)	Follow-Up Period 4 (*n* = 15237)
Continuity of care	mean (SD)	mean (SD)	mean (SD)	mean (SD)
Oncology COCI	0.71 (0.28)	0.72 (0.29)	0.71 (0.29)	0.70 (0.30)
PCP COCI	0.52 (0.31)	0.54 (0.31)	0.54 (0.31)	0.54 (0.30)
Follow-up service	%	%	%	%
Mammography ^#^	56.63	55.54	56.77	56.96
Breast Ultrasound ^#^	74.63	74.26	73.13	73.73
Mammography or Breast Ultrasound ^#^	79.89	78.49	77.55	75.28
Advanced Imaging Taking	84.75	83.62	82.95	82.18
Health Outcome	%	%	%	%
Hospitalization	13.06	10.59	10.57	10.92
Emergency Department Use	12.03	13.36	14.13	14.67

COCI: continuity of care index; PCP: primary care physician. ^#^ Excludes women with a history of bilateral mastectomy.

**Table 4:** Generalized estimating equation models for the effect of continuity of care.

Variable	Mammography			Breast Ultrasound			Advanced Imaging Test			Hospitalization			Emergency Department Use		
	OR	95% CI		OR	95% CI		OR	95% CI		OR	95% CI		OR	95% CI	
Oncology COCI ^a^	1.26	1.19	1.32	1.12	1.06	1.18	1.47	1.36	1.60	0.78	0.71	0.85	0.88	0.82	0.95
PCP COCI ^a^	1.02	0.95	1.09	0.95	0.88	1.03	1.01	0.93	1.10	0.77	0.70	0.85	0.75	0.68	0.82

COCI: Continuity of Care Index; PCP: Primary care physician; OR: Odds ratio; CI: Confidence interval. ^a^ Reference group is lower continuity of care. Using a generalized estimating equation model with binominal distribution with logit link. To avoid over-adjustment, we opted not to include in our analysis any variables that had been used in propensity score matching and had been balanced after matching.

## References

[1] Nekhlyudov L., Ganz P.A., Arora N.K., Rowland J.H. (2017). Going beyond being lost in transition: A decade of progress in cancer survivorship. J. Clin. Oncol..

[2] Institute of Medicine and National Research Council (2006). From Cancer Patient to Cancer Survivor: Lost in Transition: An American Society of Clinical Oncology and Institute of Medicine Symposium.

[3] Jacobs L.A., Palmer S.C., Schwartz L.A., DeMichele A., Mao J.J., Carver J., Gracia C., Hill-Kayser C.E., Metz J.M., Hampshire M.K. (2009). Adult cancer survivorship: Evolution, research, and planning care. CA Cancer J. Clin..

[4] Carver J.R., Shapiro C.L., Ng A., Jacobs L., Schwartz C., Virgo K.S., Hagerty K.L., Somerfield M.R., Vaughn D.J. (2007). American Society of Clinical Oncology clinical evidence review on the ongoing care of adult cancer survivors: Cardiac and pulmonary late effects. J. Clin. Oncol..

[5] Grunfeld E., Earle C.C. (2010). The interface between primary and oncology specialty care: Treatment through survivorship. JNCI Monogr..

[6] Mayer D.K., Nasso S.F., Earp J.A. (2017). Defining cancer survivors, their needs, and perspectives on survivorship health care in the USA. Lancet Oncol..

[7] Brazil K., Whelan T., O’Brien M.A., Sussman J., Pyette N., Bainbridge D. (2004). Towards improving the co-ordination of supportive cancer care services in the community. Health Policy.

[8] Lohfeld L., Brazil K., Willison K. (2007). Continuity of care for advanced cancer patients: Comparing the views of spouse caregivers in Ontario, Canada, to Dumont et al.’s theoretical model. J. Palliat. Care.

[9] Hahn E.E., Hays R.D., Kahn K.L., Litwin M.S., Ganz P.A. (2013). Use of imaging and biomarker tests for posttreatment care of early-stage breast cancer survivors. Cancer.

[10] Runowicz C.D., Leach C.R., Henry N.L., Henry K.S., Mackey H.T., Cowens-Alvarado R.L., Hurria A. (2016). American cancer society/American society of clinical oncology breast cancer survivorship care guideline. CA: Cancer J. Clin..

[11] Khatcheressian J.L., Hurley P., Bantug E., Esserman L.J., Grunfeld E., Halberg F., Hantel A., Henry N.L., Muss H.B., Smith T.J. (2012). Breast cancer follow-up and management after primary treatment: American Society of Clinical Oncology clinical practice guideline update. J. Clin. Oncol.

[12] Hahn E.E., Tang T., Lee J.S., Munoz-Plaza C.E., Shen E., Rowley B., Maeda J.L., Mosen D.M., Ruckdeschel J.C., Gould M.K. (2016). Use of posttreatment imaging and biomarkers in survivors of early-stage breast cancer: Inappropriate surveillance or necessary care?. Cancer.

[13] Baxi S.S., Kale M., Keyhani S., Roman B.R., Yang A., Derosa A., Korenstein D. (2017). Overuse of health care services in the management of cancer: A systematic review. Med. Care.

[14] Schnipper L.E., Smith T.J., Raghavan D., Blayney D.W., Ganz P.A., Mulvey T.M., Wollins D.S. (2012). American Society of Clinical Oncology identifies five key opportunities to improve care and reduce costs: The top five list for oncology. J. Clin. Oncol..

[15] Raddish M., Horn S.D., Sharkey P.D. (1999). Continuity of care: Is it cost effective. Am. J. Manag Care.

[16] Turgeon J., Dumont S., St-Pierre M., Sévigny A., Vézina L. (2006). Continuity of cancer care in Quebec: Beyond the symptoms. Can. Fam. Phys..

[17] Oeffinger K.C., McCabe M.S. (2006). Models for delivering survivorship care. J. Clin. Oncol..

[18] Masood S. (2008). Survivorship: A needed program for the continuity of care for breast cancer patients. Breast.

[19] Hudson S.V., Chubak J., Coups E.J., Blake-Gumbs L., Jacobsen P.B., Neugut A.I., Buist D.S. (2009). Identifying key questions to advance research and practice in cancer survivorship follow-up care: A report from the ASPO Survivorship Interest Group. Cancer Epidemiol. Biomark. Prev..

[20] King M., Jones L., McCarthy O., Rogers M., Richardson A., Williams R., Tookman A., Nazareth I. (2009). Development and pilot evaluation of a complex intervention to improve experienced continuity of care in patients with cancer. Br. J. Cancer.

[21] Van Walraven C., Oake N., Jennings A. (2010). The association between continuity of care and outcomes. J. Eval. Clin. Pr..

[22] Aubin M., Giguère A., Martin M., Verreault R., Fitch M.I., Kazanjian A., Carmichae P.H. (2012). Interventions to improve continuity of care in the follow-up of patients with cancer. Cochrane Database Syst. Rev..

[23] Husain A., Barbera L., Howell D., Moineddin R., Bezjak A., Sussman J. (2013). Advanced lung cancer patients experience with continuity of care and supportive care needs. Support. Care Cancer.

[24] Nazareth I., Jones L., Irving A., Aslett H., Ramsay A., Richardson A., Tookman A., Mason C., KING M. (2008). Perceived concepts of continuity of care in people with colorectal and breast cancer–A qualitative case study analysis. Eur J. Cancer Care.

[25] Lafferty J., Rankin F., Duffy C., Kearney P., Doherty E., McMenamin M., Coates V. (2011). Continuity of care for women with breast cancer: A survey of the views and experiences of patients, carers and health care professionals. Eur. J. Oncol. Nurs..

[26] Skolarus T.A., Zhang Y., Hollenbeck B.K. (2012). Understanding fragmentation of prostate cancer survivorship care: Implications for cost and quality. Cancer.

[27] Grunfeld E., Hodgson D.C., Del Giudice M.E., Moineddin R. (2010). Population-based longitudinal study of follow-up care for breast cancer survivors. J. Oncol. Pr..

[28] Lu J.F.R., Hsiao W.C. (2003). Does universal health insurance make health care unaffordable? Lessons from Taiwan. Health Aff..

[29] Cheng S.H., Hou Y.F., Chen C.C. (2010). Does continuity of care matter in a health care system that lacks referral arrangements?. Health Policy Plan..

[30] Siegel R.L., Miller K.D., Jemal A. (2016). Cancer statistics, 2016. CA Cancer J. Clin..

[31] Burstein H.J., Winer E.P. (2000). Primary care for survivors of breast cancer. N. Engl J. Med..

[32] Brenna M., Butow P., Spillane A. (2008). Survivorship care after breast cancer. Aus. Fam. Phys..

[33] Hollowell K., Olmsted C.L., Richardson A.S., Pittman H.K., Bellin L., Tafra L., Verbanac K.M. (2010). American Society of Clinical Oncology-recommended surveillance and physician specialty among long-term breast cancer survivors. Cancer.

[34] Khan N.F., Carpenter L., Watson E., Rose P.W. (2010). Cancer screening and preventative care among long-term cancer survivors in the United Kingdom. Br. J. Cancer.

[35] Lawler S., Spathonis K., Masters J., Adams J., Eakin E. (2011). Follow-up care after breast cancer treatment: Experiences and perceptions of service provision and provider interactions in rural Australian women. Support Care Cancer.

[36] Allemani C., Weir H.K., Carreira H., Harewood R., Spika D., Wang X.S., Bannon F., VAhn J., Johnson C.J., Bonaventure A. (2015). Global surveillance of cancer survival 1995–2009: Analysis of individual data for 25 676 887 patients from 279 population-based registries in 67 countries (CONCORD-2). Lancet.

[37] Chiang C.J., You S.L., Chen C.J., Yang Y.W., Lo W.C., Lai M.S. (2015). Quality assessment and improvement of nationwide cancer registration system in Taiwan: A review. Jpn. J. Clin. Oncol..

[38] Earle C.C., Neville B.A. (2004). Under use of necessary care among cancer survivors. Cancer.

[39] Snyder C.F., Frick K.D., Herbert R.J., Blackford A.L., Neville B.A., Carducci M.A., Earle C.C. (2011). Preventive care in prostate cancer patients: Following diagnosis and for five-year survivors. J. Cancer Surviv..

[40] Snyder C.F., Frick K.D., Herbert R.J., Blackford A.L., Neville B.A., Wolff A.C., Carducci M.A., Earle C.C. (2013). Quality of care for comorbid conditions during the transition to survivorship: Differences between cancer survivors and noncancer controls. J. Clin. Oncol..

[41] Pollack C.E., Frick K.D., Herbert R.J., Blackford A.L., Neville B.A., Wolff A.C., Carducci M.A., Earle C.C., Snyder C.F. (2014). It’s who you know: Patient-sharing, quality, and costs of cancer survivorship care. J. Cancer Surviv..

[42] Bice T.W., Boxerman S.B. (1977). A quantitative measure of continuity of care. Med. Care.

[43] Smedby Ö., Eklund G., Eriksson E.A., Smedby B. (1986). Measures of continuity of care: A register-based correlation study. Med. Care.

[44] Earle C.C., Burstein H.J., Winer E.P., Weeks J.C. (2003). Quality of non–breast cancer health maintenance among elderly breast cancer survivors. J. Clin. Oncol..

[45] Snyder C.F., Earle C.C., Herbert R.J., Neville B.A., Blackford A.L., Frick K.D. (2008). Preventive care for colorectal cancer survivors: A 5-year longitudinal study. J. Clin. Oncol..

[46] Snyder C.F., Frick K.D., Peairs K.S., Kantsiper M.E., Herbert R.J., Blackford A.L., Wolff A.C., Earle C.C. (2009). Comparing care for breast cancer survivors to non-cancer controls: A five-year longitudinal study. J. Gen. Intern. Med..

[47] Mainous A.G., Gill J.M. (1998). The importance of continuity of care in the likelihood of future hospitalization: Is site of care equivalent to a primary clinician?. Am. J. Public Health.

[48] Gill J.M., Mainous A.G., Nsereko M. (2000). The effect of continuity of care on emergency department use. Arch. Fam. Med..

[49] Chu H.Y., Chen C.C., Cheng S.H. (2012). Continuity of care, potentially inappropriate medication, and health care outcomes among the elderly: Evidence from a longitudinal analysis in Taiwan. Med. Care.

[50] Deyo R.A., Cherkin D.C., Ciol M.A. (1992). Adapting a clinical comorbidity index for use with ICD-9-CM administrative databases. J. Clin. Epidemiol..

[51] Austin P.C. (2011). An introduction to propensity score methods for reducing the effects of confounding in observational studies. Multivar. Behav. Res..

[52] Ghisletta P., Spini D. (2004). An introduction to generalized estimating equations and an application to assess selectivity effects in a longitudinal study on very old individuals. J. Educ. Behav. Stat..

[53] Shin D.W., Kim Y.W., Oh J.H., Kim S.W., Chung K.W., Lee W.Y., Lee J.E., Lee W.C., Guallar E., Cho J. (2011). Knowledge, attitudes, risk perception, and cancer screening behaviors among cancer survivors. Cancer.

[54] Field T.S., Doubeni C., Fox M.P., Buist D.S.M., Wei F., Geiger A.M., Quinn V.P., Lash T.L., Prout M.N., Yood M.U. (2007). Under utilization of surveillance mammography among older cancer survivors. J. Gen. Intern. Med..

[55] Snyder C.F., Frick K.D., Kantsiper M.E., Peairs K.S., Herbert R.J., Blackford A.L., Wolff A.C., Earle C.C. (2009). Prevention, screening, and surveillance care for breast cancer survivors compared with controls: Changes from 1998 to 2002. J. Clin. Oncol..

[56] Virgo K.S., Lerro C.C., Klabunde C.N., Earle C., Ganz P.A. (2013). Barriers to breast and colorectal cancer survivorship care: Perceptions of primary care physicians and medical oncologists in the United States. J. Clin. Oncol..

[57] Hong J.S., Kang H.C., Kim J. (2010). Continuity of care for elderly patients with diabetes mellitus, hypertension, asthma, and chronic obstructive pulmonary disease in Korea. J. Korean Med. Sci..

[58] Hussey P.S., Schneider E.C., Rudin R.S., Fox D.S., Lai J., Pollack C.E. (2014). Continuity and the costs of care for chronic disease. JAMA Intern. Med..

[59] Lin I.P., Wu S.C. (2015). The Determinants of Continuity of Care for Patients with Chronic Obstructive Pulmonary Disease. Taiwan J. Public Health.

[60] Ryvicker M., Russell D. (2018). Individual and Environmental Determinants of Provider Continuity Among Urban Older Adults with Heart Failure: A Retrospective Cohort Study. Gerontol. Geriatr. Med..

[61] Simard S., Savard J. (2009). Fear of cancer recurrence inventory: Development and initial validation of a multidimensional measure of fear of cancer recurrence. Support. Care Cancer.

[62] Cree M., Bell N.R., Johnson D., Carriere K.C. (2006). Increased continuity of care associated with decreased hospital care and emergency department visits for patients with asthma. Dis. Manag..

[63] Menec V.H., Sirski M., Attawar D., Katz A. (2006). Does continuity of care with a family physician reduce hospitalizations among older adults?. J. Health Serv. Res. Policy.

[64] Ionescu-Ittu R., McCusker J., Ciampi A., Vadeboncoeur A.M., Roberge D., Larouche D., Verdon J., Pineault R. (2007). Continuity of primary care and emergency department utilization among elderly people. Can. Med. Assoc. J..

[65] Worrall G., Knight J. (2011). Continuity of care is good for elderly people with diabetes: Retrospective cohort study of mortality and hospitalization. Can. Fam. Phys..

[66] Huang S.T., Wu S.C., Hung Y.N., Lin I.P. (2016). Effects of continuity of care on emergency department utilization in children with asthma. Am. J. Manag/ Care.

[67] Parchman M.L., Pugh J.A., Noël P.H., Larme A.C. (2002). Continuity of care, self-management behaviors, and glucose control in patients with type 2 diabetes. Med. Care.

[68] Chen C.C., Tseng C.H., Cheng S.H. (2013). Continuity of care, medication adherence, and health care outcomes among patients with newly diagnosed type 2 diabetes: A longitudinal analysis. Med. Care.

[69] Cook R.I., Render M., Woods D.D. (2000). Gaps in the continuity of care and progress on patient safety. BMJ.

[70] Guilcher S.J., Hogan M.E., Calzavara A., Hitzig S.L., Patel T., Packer T., Lofters A.K. (2018). Prescription drug claims following a traumatic spinal cord injury for older adults: A retrospective population-based study in Ontario, Canada. Spinal Cord.

